# Are the standards of professionalism expected in dentistry justified? Views of dental professionals and the public

**DOI:** 10.1038/s41415-023-5572-8

**Published:** 2023-03-10

**Authors:** Hannah Barrow, Sophie Bartlett, Alison Bullock, Jonathan Cowpe

**Affiliations:** 41415173642001 NIHR Academic Clinical Fellow in Special Care Dentistry, Eastman Dental Institute, University College London, UK; 41415173642002 Cardiff Unit for Research and Evaluation in Medical and Dental Education, School of Social Sciences, Cardiff University, UK; 41415173642003 School of Dentistry, Cardiff University, UK

## Abstract

**Introduction** In the UK, the General Dental Council specifies nine principles of professional standards that dental registrants must follow. There are views that such standards are high, patients' expectations are rising, and the professionalism of dental professionals is increasingly scrutinised. This paper explores whether the high standards expected in dentistry are justified.

**Methods** We applied thematic analysis to 772 free-text responses from dental team members and the public to a modified Delphi survey. Respondents described their views of professional and unprofessional behaviours in dentistry. Data were obtained as part of a larger review of professionalism in dentistry.

**Results** Two lines of argument were identified: professionalism standards are high, but justifiably so; and professionalism standards are too high. Within these, four broad themes emerged: patient trust; comparison with other professions; a culture of fear; and perfection.

**Conclusion** High professionalism standards are justified in a profession where patient trust is paramount. However, a problem lies in the culture that surrounds professionalism in terms of litigation and dental professionals feel pressure to possess an unattainable, infallible nature. These negative impacts need minimising. We suggest that undergraduates and continuing professional development approach professionalism with care, to foster a supportive, positive and reflective culture of professionalism.

## Key points

- Highlights the complexities surrounding whether the standards of professionalism in dentistry are justified.
- Highlights that high professionalism standards are expected in a profession where patient trust is paramount; however, dental professionals feel pressure to possess an unattainable infallible nature and fear increasingly high levels of litigation.
- Suggests that undergraduate and continuing professional development aim to foster a supportive, positive, and reflective culture of professionalism to help prevent a culture of fear contributing to defensive dentistry.

## Introduction

To practise as a dentist or dental care professional (DCP) in the UK requires registration with the General Dental Council (GDC). As part of this registration, the GDC sets professional standards for the dental team in areas of conduct, performance and ethics.^1^ There are nine principles that registrants are expected to follow 'at all times' and reflect 'what patients can expect from the dental team'.^1^

Professionalism is underpinned by the 'qualities connected with trained and skilled people';^2^ however, a straightforward definition is somewhat elusive.^3^ Professionalism is a broad, complex concept, open to interpretation and lacks clarity on how a breach or lapse in professionalism might be defined.^4^^,^^5^ The concept encompasses many facets, including technical ability, as well as human qualities, behaviours and values.^3^^,^^6^

 Over the last several decades, 'the patient experience' has been a focus of discussion in healthcare settings.^3^ Within the dentistry profession, there is a view that standards expected of dental professionals, specifically by the public and regulatory body, are high.^7^ Patients' expectations of the quality of dental care and service delivery are not only rising, but deviating from the expectations within the profession.^8^^,^^9^ Dissatisfaction, challenge and sometimes, litigation, can arise where there is disparity between patient and professional interpretation of professionalism^6^^,^^9^ and between patients' expectations and what is feasible in routine practice. The impact of this can be detrimental. Research demonstrates that regulation, litigation and patient interaction are some of the most common reasons for burnout and mental health issues among dentists.^10^

A review of professionalism undertaken under the auspices of the Association for Dental Education in Europe (ADEE) was published in 2020.^7^ This paper aims to build on that report by reviewing in more detail data related to the question of whether the high standards expected of dental professionals by both the profession and the public are justified.

## Methods

The ADEE team used a modified online Delphi survey to investigate professionalism and reach consensus on the boundaries of acceptable behaviour for those working in dentistry.^7^^,^^11^^,^^12^ Participants were presented with questions derived from a review of existing literature and evidence collected through scoping interviews with topic experts and focus groups with dentists, DCPs and members of the public. The survey was disseminated through deans of dental schools, royal colleges, postgraduate deans and directors and distributed via social media platforms (Twitter and Facebook) to reach a wider public audience.

The survey statements were divided into two groups: 'unprofessional' (negative) behaviours and 'professional' (positive) behaviours. Respondents were requested to designate whether a 'professional' behaviour statement was 'essential', 'desirable' or 'not necessary'. The 'unprofessional' behaviour statements were rated as 'highly', 'moderately', or 'not' unprofessional. Consensus was deemed to be achieved when 70% of respondents agreed on the rating of that statement. Two rounds were conducted. Round one was open between 13 November 2019 and 30 November 2019. Results were aggregated and round two was open from 9 December 2019 until 24 December 2019. In the second round, respondents were asked to reconsider their responses in the light of aggregated results, for items where consensus was not achieved. The numeric results are given in the ADEE report^7^ but respondents were also invited to provide comment on the statements. This exercise produced a vast amount of additional data which was not included in the report.^7^ This paper reports on the analysis of responses provided in round one to three open questions that invited commentary on unprofessional behaviours (312 responses), professional behaviours (216 responses), and any other reflections around professionalism in dentistry (244 responses). In total, 33,976 words of free-text data were yielded.

All open-text responses were transferred into NVivo for pattern coding and analysis. Analysis was thematic.^13^ Codes were identified by authors HB and SB and discussed with AB and JC. In reporting extracts, we indicate (in brackets) the respondent's self-reported profession (dentist; DCP; educator; policymaker or regulator; member of the public). Ethical approval for this research was obtained from the School of Social Sciences at Cardiff University (SREC/3390). Consent to participate was implied by completion of the survey and no individual is identifiable from any data we report.

## Results

**Fig. 1:**
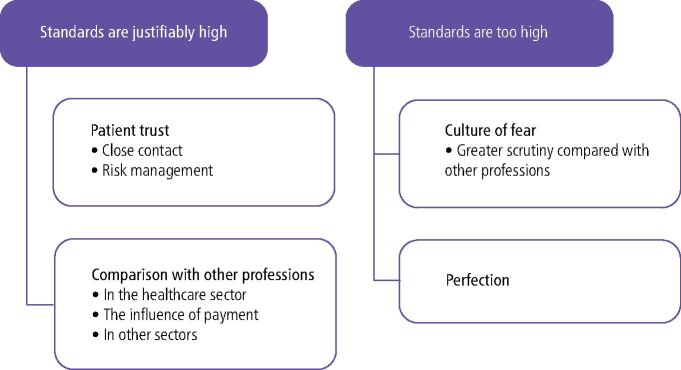
Thematic overview

Perceptions of professionalism in dentistry fell into one of two main arguments: that standards of professionalism are high, but justifiably so; or that standards of professionalism are too high. Within these lines of argument, we identified themes and subthemes, summarised in Figure 1.

We report on each of these themes in the context of the two primary lines of argument, noting that some themes are cross-cutting.

### Standards are justifiably high

#### Patient trust

There is a perception that the high standards required of dental professionals are necessary to enable patient trust. Assessment and treatment of the oral cavity necessitates close contact between the clinician and patient in a personal and vulnerable area of the body and is known to cause anxiety and fear of pain.^14^ Thus, respondents argued that to build trust, inspire confidence and address 'widespread dental fear and phobia' (dentist), it is vital to implement 'rigorous professional standards'. Others explained:

- 'Because of the nature of the job we do and the close contact we have with patients while operating in their mouths, I believe the standards of professionalism expected are higher [than other professions]' (dentist)
- 'Given that patients are unusually fearful of the dentist in comparison with other medical professionals, the onus of building trust is more profound…we need to conform to higher standards of professionalism because of this' (dentist).

Patient trust was also seen as critical for a profession requiring risk management. Risks apply to all dental treatments and decisions, from irreversible interventions to deciding to leave and monitor. These risks can be harmful and substantial, for example, paraesthesia, bleeding and infection:^15^

- 'I think it [professionalism] relates to how invasive the interaction is, and how much risk and therefore trust is involved in the care' (policymaker/regulator).

Irreversible procedures, such as tooth extraction or removal of tooth surfaces, can have a major impact on a patient's life^16^ and thus it was argued high standards of professionalism are to be expected:

- 'There is a greater expectation placed on being able to be at the "top of your game" when, for example, undertaking irreversible procedures' (policymaker/regulator).

The performance of such procedures demands a combination of skills that were seen to justify the high standards of professionalism in dentistry:

- 'No other profession requires the same combination of technical skill, clinical knowledge and clinical judgement in one individual' (public).

#### Comparisons with other professions

Respondents drew comparisons between the standards expected in dentistry and a number of other professions. In the context of other healthcare professions, particularly medicine, many perceived that standards of professionalism were similar to those in dentistry. This was reasoned to be due to the high level of risk, the invasiveness of the actions and the need for trust:

- 'Dentistry would be equivalent with a medical profession…largely because our actions or inactions can lead to harm' (DCP and educator)
- 'The motivation for continuous improvement in skill and knowledge is needed in dentistry, probably more than in any other profession, with the possible exception of medicine and surgery' (public).

However, this view was not unanimous, and some felt that dental professionals were held to a higher standard than medical professionals. On these occasions, money was commonly mentioned. In contrast to NHS medical treatment, NHS dental treatment often necessitates a financial cost to the patient, unless they meet exemption criteria. As a result, some maintained that this shifts the dynamic between patient and healthcare provider and dental patients increasingly view themselves as 'consumers' with a concomitant rise in expectations:

- 'Patients often see themselves as consumers, as opposed to receiving healthcare, and so the relationship between a GDP and their patient very much differs to that of their GP' (dentist).

The image of dentistry as a high earning profession was another reason for believing that higher standards are expected. This was seen by some as fostering a reduction in respect that the public accord to dentists and was emphasised when comparisons were made to nurses:

- 'I have seen comments from patients on Twitter such as "with the amount I pay my dentist, they should be available to take my call in the middle of the night" etc. I think this places a different standard expectation [compared] with other professions, such as nursing staff who are known to be paid less, and perceived to be stretched more in terms of staffing levels etc' (dentist).

Comparisons were also drawn between expectations of professionalism in dentistry and a number of non-healthcare professions. Here, participants' opinions varied. Some viewed healthcare professions as being 'held to higher standards than, for example, solicitors, police officers, accountants or politicians' (dentist). The potential longevity of the dentist-patient relationship was posed as one reason for high standards of professionalism that might outstrip the expectations placed on others:

- 'Patients often have longer relationships with dentists than other professionals, especially in small communities and so the distance between dentist and patient can become closer than with a lawyer you see once every few years in the city' (dentist).

Others perceived dentistry standards of professionalism like other professions, such as veterinarians, the police and religious leaders. Trust was seen as just as important within these roles and professionalism necessary to reflect and develop that confidence:

- 'Trust, confidence, behaviour etc should be the same whether the individual works in medical, veterinary, police, religion etc fields' (dentist).

Another view was that standards of professionalism should be the same for any profession that 'look after and help members of the public' (dentist).

### Standards are too high

#### Culture of fear

Some reflected that although standards of professionalism may be similar across professions, compared to others, they felt there was greater scrutiny and judgement in dentistry:

- 'There seems to be a scrutiny of dental professionals particularly, or we are more keenly aware of it due to the litigation culture' (DCP).

This was seen as feeding a culture of fear. Advertisements for litigation action and films portraying dentists as 'demons'^17^ were reported:

- 'The image of dentistry is not great and that is as much as anything else down to portrayal in the media, by the film industry…' (dentist and educator).

Not only did this result in some feeling the need to exhibit very high standards of professionalism to 'disprove' this negative image, but also that fear of litigation might sometimes prevent dentists providing certain treatments. Undergraduate education, rather than mitigating a culture of fear, was seen to exacerbate such feelings. Some spoke of professionalism lapses being discussed at university in an alarming manner:

- 'I feel like it is drilled into us at university and although I do agree it is very important, I do think in certain areas it is too extreme' (DCP)
- 'Care needs to be taken to not create or even extend a culture of fear acting on the profession that is ultimately detrimental to patients. I have already seen young dentists avoiding even trying treatments under supervision for fear of the consequences if it goes wrong and ends up at the GDC' (dentist and educator).

#### Perfection

Some respondents felt that dental professionals were portrayed as expected to possess an infallible nature, unable to make mistakes. These respondents argued that to meet such standards would require dental professionals to be 'robots', 'saints', or 'perfect' human beings:

- 'It's totally unfair that dentists/dental professionals are expected to act like perfect robotic humans' (dentist)
- 'Dental professionals should be held to a good standard of behaviour but they should not be expected to be saintly at all times. This is an unreasonable position' (dentist).

Comments were also made that dental professionals were not well-prepared for when things do go wrong and this in turn contributed to a sense that the standards of professionalism were unachievable and unrealistic:

- 'The bar is set very high. We have been trained that nothing should ever go wrong in healthcare - this is not realistic' (dentist).

## Discussion

The results demonstrate the high standards of professionalism that the study participants (members of the dental team, as well as the public) perceive as expected in dentistry. Attending a dental appointment is associated with much higher anxiety levels than other healthcare appointments and consequently, trust is frequently hard-won from patients. Dental anxiety is prevalent among the general public and 12% of adults have been classified as having extreme dental anxiety.^14^ Our study shows how high standards of professionalism are seen by many to be necessary to reassure patients and reduce anxiety among those who may have had previous negative experiences.

Nonetheless, many dentist respondents in particular feel under pressure to be 'perfect', which is essentially unattainable. In a profession where the relationship between the patient and the dentist relies on excellent lines of communication and trust, if dentists are expected to be infallible, there is a risk that they may avoid difficult questions and treatments for fear of adverse reactions from patients. On a broad scale, a UK dentist has a strong likelihood of being subject to a negligence claim once a decade, a frequency that exceeds that of medical general practitioners.^6^ The culture of litigation has grown in dentistry: a Dental Protection survey^18^ revealed that 89% of respondents were fearful of being sued by patients and of those, 74% felt that this fear impacts the way they work. The practice of 'defensive dentistry' results in a move away from more desirable 'patient-centred' care. Those working in dentistry who perceive standards of professionalism to be too high may respond defensively and see any criticism or lapse in professionalism as a personal attack rather than an opportunity to reflect and develop.

Advertisements for dental litigation, stories about extreme cases of dental misconduct and portrayal of dentists as greedy put the dental workforce under pressure to disprove this image. In this way, the media image of dentistry contributes to a sense of overly high standards of professionalism. The impact of such pressure on mental health and the enjoyment of work is potentially significant, unwelcome, and likely exacerbated by working in relative isolation; most dentists work alone in a surgery with a dental nurse. This contrasts with hospital-based doctors, for example, who often work as a part of a large team.

The shift to patients seeing themselves as 'consumers' in the dentistry 'business' brings with it public expectations of especially high standards of professionalism, as they pay for the care they receive. As a result, dental professionals in our study contrasted dentistry with medicine and expressed a sense of heightened pressure to live up to dental patients' expectations. By contrast, however, there was evidence that the public perceive the standards of professionalism in dentistry to be similar to other professions and not just those in healthcare. Such results could suggest that it is not the height of standards that are problematic in dentistry, but the culture that surrounds professionalism and the overwhelming threat of litigation.

Undergraduate education and continuing professional development (CPD) provide important opportunities to influence the perceptions of standards of professionalism. Dental professionals need guidance in distinguishing the sense of duty and responsibility appropriate to a professional from fear of litigation and defensive practice. This is important throughout all career stages of dentistry. Those in the early stages of their career are shaped and influenced by those they encounter as role models and a defensive approach will be transmitted to the newer recruits and graduates and affect attitudes to work. 

Developing an ethos of support can help ease a sense of performance to unattainable standards of perfection. It is important to promote a positive attitude to professionalism, encouraging reflection and lifelong learning. Mentoring could help guide dental professionals in how to best maintain professionalism and strive for improvement for good reasons: to provide the best possible care and outcome for their patients.

## Conclusion

Although only a snapshot of the views, the results show that across all respondent groups, high standards of professionalism are expected in dentistry and stem from the importance of engendering patient trust in a profession requiring close contact and at times, invasive treatment. While our results suggest that these high standards are justified, the negative culture that surrounds professionalism and widespread fears of increasing litigation need addressing. At present, this culture of fear underpins a defensive approach that undermines dental professionals' approach to patient care, as well as their own wellbeing and job satisfaction. We therefore contend that although there are sound reasons for high standards of professionalism in dentistry, the negative impact needs to be minimised for both the sake of dental professionals and patients.

We suggest that care is needed in how professionalism is approached in undergraduate and CPD settings to ensure that learners feel well-supported and able to develop a positive and constructive approach to professionalism. The data signpost the need for dental professionals to develop the skills to learn and reflect throughout their careers to strive towards maintaining achievable professionalism standards. How dental professionals may receive support and from whom (peers, teams, educators, and the regulatory body) is worthy of further study.

## References

[1] General Dental Council. Standards for the Dental Team. 2019. Available at https://www.gdc-uk.org/standards-guidance/standards-and-guidance/standards-for-the-dental-team (accessed December 2022).

[2] Cambridge University Press. *Cambridge Academic Content Dictionary*. Cambridge: Cambridge University Press, 2009.

[3] Tallis R C. Doctors in society: medical professionalism in a changing world. *Clin Med (Lond) *2006; **6:** 7-12. 10.7861/clinmedicine.6-1-7 PMC4954437 16521348

[4] Trathen A, Gallagher J E. Dental professionalism: definitions and debate. *Br Dent J* 2009; **206:** 249-253. 10.1038/sj.bdj.2009.164 19287419

[5] General Dental Council. General Dental Council Patient and Public Survey 2017. 2017. Available at https://www.gdc-uk.org/docs/default-source/research/public-and-patient-2017-topline.pdf?sfvrsn=70bfcdb7_2 (accessed December 2022).

[6] Lewis K. Professionalism - A Medico-Legal Perspective. *Prim Dent J* 2021; **10:** 51-56. 10.1177/20501684211018573 34353164

[7] General Dental Council*.* Professionalism: A Mixed-Methods Research Study. 2020. Available at https://www.gdc-uk.org/about-us/what-we-do/research/our-research-library/detail/report/professionalism-a-mixed-methods-research-study (accessed February 2023).

[8] Zijlstra-Shaw S, Robinson P G, Roberts T. Assessing professionalism within dental education; the need for a definition. *Eur J Dent Educ* 2011; **16:** 128-136. 10.1111/j.1600-0579.2011.00687.x 22251336

[9] Taibah S M. Dental professionalism and influencing factors: patients' perception. *Patient Prefer Adherence* 2018; **3:** 1649-1658. 10.2147/PPA.S172788 PMC6126481 30214167

[10] British Dental Association. The Mental Health and Well-being of UK Dentists: A Qualitative Study. 2017. Available at https://www.bda.org/about-the-bda/campaigns/Documents/The%20Mental%20Health%20and%20Well-being%20of%20UK%20Dentists.pdf (accessed December 2022).

[11] Powell C. The Delphi technique: myths and realities. *J Adv Nurs *2003; **41:** 376-382. 10.1046/j.1365-2648.2003.02537.x 12581103

[12] Duffield C. The Delphi technique: a comparison of results obtained using two expert panels. *Int J Nurs Stud* 1993; **30:** 227-237. 10.1016/0020-7489(93)90033-q 8335432

[13] Braun V, Clarke V. Using Thematic Analysis in Psychology. *Qual Res Psychol *2006; **3:** 77-101.

[14] NHS Digital. Adult Dental Health Survey 2009. 2011. Available at https://digital.nhs.uk/data-and-information/publications/statistical/adult-dental-health-survey/adult-dental-health-survey-2009-summary-report-and-thematic-series (accessed December 2022).

[15] NHS. Complications. 2021. Available at https://www.nhs.uk/conditions/wisdom-tooth-removal/complications/ (accessed December 2022).

[16] Davis D M, Fiske J, Radford D R. The emotional effects of tooth loss: a preliminary quantitative study. *Br Dent J* 2000; **188:** 503-506. 10.1038/sj.bdj.4800522 10859849

[17] Walliams D. *Demon Dentist*. London: HarperCollins, 2013.

[18] Dental Protection. Dental Protection survey reveals 9 in 10 dentists fear being sued by patients. 2018. Available at https://www.dentalprotection.org/uk/articles/dental-protection-survey-reveals-9-in-10-dentists-fear-being-sued-by-patients (accessed December 2022).

